# Development and Evaluation of Vibration Canceling System Utilizing Macro-Fiber Composites (MFCs) and Long Short-Term Memory (LSTM) Vibration Prediction AI Algorithms for Road Driving Vibrations

**DOI:** 10.3390/ma17102299

**Published:** 2024-05-13

**Authors:** Sang-Un Kim, Joo-Yong Kim

**Affiliations:** 1 Department of Smart Wearable Engineering, Soongsil University, Seoul 06978, Republic of Korea; tkddnsl0723@naver.com; 2 Department of Materials Science and Engineering, Soongsil University, Seoul 06978, Republic of Korea

**Keywords:** mitigating driving vibrations, active vibration cancellation system (AVC system), macro-fiber composite (MFC), long short-term memory recurrent neural network (LSTM RNN), vibration prediction algorithms

## Abstract

This study developed an innovative active vibration canceling (AVC) system designed to mitigate non-periodic vibrations during road driving to enhance passenger comfort. The macro-fiber composite (MFC) used in the system is a smart material that is flexible, soft, lightweight, and applicable in many fields as a dual-purpose sensor and actuator. The target vibrations are road vibration data that were collected while driving on standard urban (Seoul) and highway roads at 40 km/s. To predict and cancel the target vibration accurately before passing it, we modeled the vibration prediction algorithm using a long short-term memory recurrent neural network (LSTM RNN). We regenerated vibrations on Seoul and highway roads at 40 km/s using MFCs and measured the displacements of the measured, predicted, and AVC vibrations of each road condition. To evaluate the vibration, we computed the root mean squared error (RMSE) and compared standard deviation (SD) values. The accuracies of LSTM RNN vibration prediction algorithms are 97.27% and 96.36% on Seoul roads and highway roads, respectively, at 40 km/s. Although the vibration ratio compared with the AVC results are different, there was no difference between the values of the AVC vibrations. According to a previous study and the principle of the AVC system, the target vibrations decrease by canceling the inverse vibration of the MFC actuator.

## 1. Introduction

Smart materials are substances that respond to changes or impacts in external environmental factors such as light, temperature, humidity, electricity, and pressure, thereby altering their properties. Generally, these materials exhibit changes in mechanical, optical, and electrical characteristics. Among the various types of smart materials, piezoelectric materials are notable for exhibiting the piezoelectric effect [1,2,3,4], where their electrical properties change in response to pressure, and for possessing reversible characteristics known as the inverse piezoelectric effect [5,6,7,8], where the application or alteration of an electric field induces deformation.

Due to their reversible nature, piezoelectric materials are utilized not only as sensors for measuring pressure but also as actuators capable of generating pressure [9,10,11], vibration, and sound [12] through electrical signals, distinguishing them from other smart materials. Particularly, there has been extensive research utilizing piezoelectric materials for vibration-related studies. In one study, a vibration sensor module utilizing PZT piezoelectric material in the form of microelectromechanical systems (MEMS) was fabricated and evaluated [13]. The results showed higher sensitivity compared to other sensors using resistive or capacitive methods. In a study related to actuators, a transparent haptic device was developed by applying an alternating voltage to PZT film, enabling the creation of actuators capable of sensing subtle changes in friction [14].

Lead zirconate titanate (PZT), polyvinylidene fluoride (PVDF), barium titanate, scandium powder, and abaite are among the most notable piezoelectric materials. The piezoelectric effect is particularly pronounced in ceramic materials with perovskite crystal structures [15,16,17]. This is because the arrangement of metal atoms in these materials is inherently asymmetrical, making them prone to developing electrical polarization when subjected to deformation. Despite their remarkable piezoelectric properties, traditional piezoelectric ceramic materials have limited flexibility. In response to these limitations, new composites are being developed with piezoelectric materials, such as PZT fibers used in MFCs [18,19]. These composites provide greater flexibility [20] and displacement capabilities [21,22].

The MFC can be a revolutionary composite sensor or actuator that was recently developed at the NASA Langley Research Center [18]. An MFC consists of piezoelectric fibers arranged in an epoxy matrix. Due to their composite structure, they have the flexibility suitable for curved surfaces, making them applicable for vibration control and structural health monitoring applications [23]. Furthermore, in vibration control research, MFCs are capable of simultaneously functioning as sensors and actuators. They can generate voltage without requiring external power sources. Additionally, due to the immediate deformation of their crystal structure, they offer very fast sensing and actuating response speeds [24,25,26].

Prior research related to vibration control conducted a comparison between passive gels, which constantly absorb vibrations, and an active vibration cancellation (AVC) utilizing MFCs for vibration sensing while simultaneously canceling external vibrations with reverse-phase vibrations. This comparison aimed to assess the vibration attenuation performance and applicability of the systems [27].

Motion sickness is a significant issue among passengers in vehicles, causing symptoms such as dizziness, nausea, vomiting, and reduced concentration, leading to unpleasantries [28,29]. Some studies suggest that the vibrations within vehicles, stemming from various sources such as the engine, steering, tires, and other components, can induce motion sickness and discomfort among passengers [30,31].

In this study, we aimed to investigate the practical application of AVC with embedded MFCs for attenuating vibrations occurring during vehicle driving. We collected vibration data using MFC sensors while driving an actual vehicle on roads, targeting specific conditions where vibrations occur. 

To effectively cancel the collected non-periodic vibrations with rapid attenuation, we developed a vibration prediction algorithm using an artificial intelligence (AI) algorithm. There is a long short-term memory (LSTM) recurrent neural network (RNN) AI algorithm which is a kind of deep learning. LSTM RNN has seen widespread recent use with strong results in prediction and in overcoming the problems of RNN, such as vanishing or exploding gradients, during the training process through backpropagation. As a study in the development of vibration prediction algorithms using LSTM RNN, we developed a system capable of providing warnings based on predictive algorithms created from data obtained from aircraft experiencing excessive vibrations [32]. 

Finally, we evaluated the performance of AVC through simulated experiments by assessing the attenuation performance using actual driving vibrations and AVC signals obtained from the prediction algorithm.

## 2. Materials and Methods

### 2.1. MFC Sensor, Actuator, and Active Vibration Cancleation (AVC)

**Figure 1:**
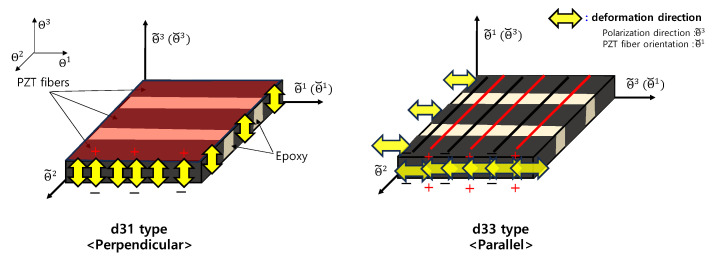
The 3 coordinate systems for d31 and d33 types of MFCs.

In MFCs, depending on the arrangement of the PZT fibers and the direction of the piezoelectric effect, they are divided into perpendicular d31 and parallel d33 types. These types are designed by optimizing the electric field for each type according to the electrode shape and arrangement. In MFC research, three coordinate systems, the curvilinear coordinate system, fiber coordinate system, and polarization coordinate system, are respectively denoted by $\Theta^{i},\;{\overset{˘}{\Theta }}^{i},\;{\tilde{\Theta }}^{i}\;\left(i=1,2,3\right),$ and they are used to describe the d31 and d33 types as shown in Figure 1. The vibration of driving occurs in the Z direction, which corresponds to the $\Theta^{3}$ direction in the coordinate system. Therefore, to cancel the vibration, a d31 type, where the direction of the force exerted by the PZT fibers matches that of the $\Theta^{3}$ direction, is suitable. 

**Figure 2:**
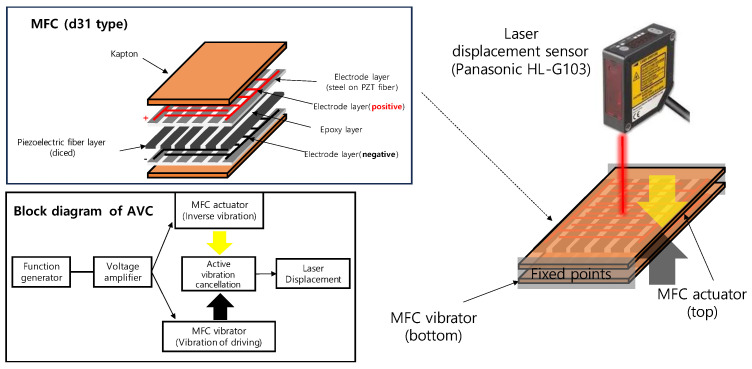
The MFC structure and experimental setup of AVC.

In this study, we used a d31 type of MFC sensor and actuator made from Smart Material Corp [19]. The MFC is packaged with Kapton layers at the top and bottom, as shown in Figure 2. Between the cathode and anode layers, there are diced PZT fibers embedded in epoxy composite layers. The PZT fibers in the MFC sensor can generate the voltage when external forces or vibrations deform PZT fibers by piezoelectric effects, and the PZT fibers in the MFC actuator can stretch or contract by the electric field.

The mechanical and electronical characteristics associated with this MFC are summarized in Table 1, which presents the properties provided by the Smart Material Corp [19]. The piezoelectric effect and inverse piezoelectric effect of the MFC sensor and actuator can be represented by the constitutive equations shown in Equations (1) and (2) [33].

(1) $$\overset{˘}{\varepsilon }=\overset{˘}{s}\overset{˘}{\sigma }+\overset{˘}{d}\overset{˘}{E}$$

(2) $$\overset{˘}{D}={\overset{˘}{d}}^{T}\overset{˘}{\sigma }+\overset{˘}{\epsilon }\overset{˘}{E}$$

where the $\overset{˘}{\varepsilon }$ is the linearized strain, $\overset{˘}{s}$ is the elastic compliance, $\overset{˘}{\sigma }$ is the stress, $\overset{˘}{d}$ is the piezoelectric constant, ${\overset{˘}{d}}^{T}$ is the inverse piezoelectric constant (transposed piezoelectric constant), $\overset{˘}{E}$ is the electric field strength, $\overset{˘}{D}$ is the electric displacement, and $\overset{˘}{\epsilon }$ is the permittivity. Equation (1) above summarizes the relationship between the force generated by the linear deformation of PZT fibers and the change in intensity of the electric field, explaining the piezoelectric effect. Equation (2) describes the converse piezoelectric effect, where deformation is induced by applying an electric field or voltage. 

The MFC layer of the d31 type used in this study consists of Kapton layers at the top and bottom, electrode layers, and a central structure containing diced PZT fibers. The AVC operates with both ends of two MFCs fixed. In this setup, the bottom MFC acts as a vibrator, generating vibrations that occur during driving, as shown in Figure 2. The role of the top MFC is to generate vibrations of the inverse phase to cancel out the vibrations within operation voltage range. 

**Figure 3:**
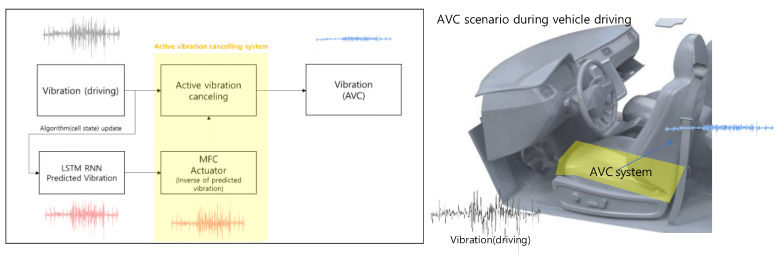
The block diagram of the total system and AVC scenario.

In the final vibration-cancelling system of this study, driving vibrations are measured concurrently with predicted vibrations generated by an LSTM RNN algorithm. Subsequently, the algorithm undergoes updates, and the predicted vibrations are used to cancel the driving vibration after being inversed to input to the MFC actuator. One of the AVC system scenarios is the car seat canceling the driving vibration to mitigate motion sickness, as shown in Figure 3.

### 2.2. LSTM (Long Short-Term Memory) RNN (Recurrent Neural Network) Vibration Prediction Algorithm Based on Driving Vibration Data

In this study, the LSTM used obtained displacement data based on normalized vibration accelerometer data by conducting road driving tests on a real vehicle (Hyundai, GV 70, Ulsan, Republic of Korea) on standard roads in Seoul and on the highway, respectively, running at 40 km/h. The accelerometer was attached to the actual car seat’s buttock area, with an 80 kg passenger seated. The sampling frequency, the resolution, and range are 256 Hz, 0.488 milli gravitational acceleration (mg), and 16 g, respectively.

**Figure 4:**
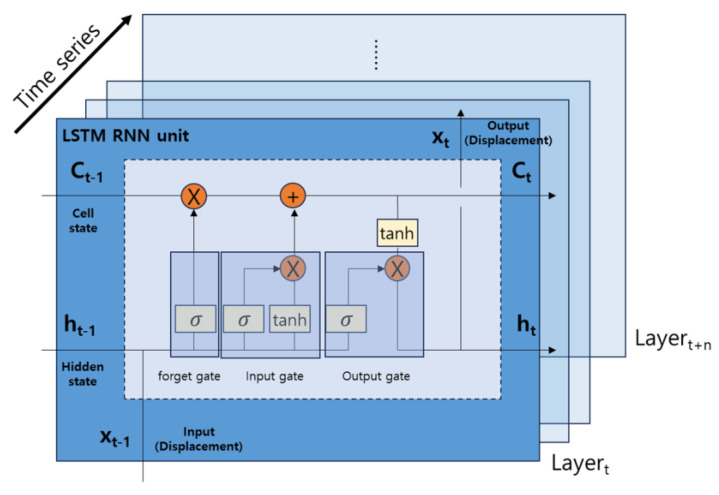
LSTM RNN vibration prediction algorithm.

The modeling of the LSTM RNN’s vibration prediction algorithm is depicted in Figure 4. Displacement data *X_t_*_−1_ representing predicted time-dependent driving vibration is fed as input data, and, through cells that retain information from previous data, *X_t_* is output as the predicted displacement data for the next time step. In this process, the state of the cell (*C_t_*) represents the overall processing structure over time, and the hidden state (*h_t_*) within this cell manages memory and updates. Specifically, each layer of the LSTM RNN updates its cell state through the forget gate, input gate, cell gate, and output gate. The processes for each gate can be explained by the following Equations (3)–(6).

(3) $$i_{t}=\sigma_{g}\left(W_{i}X_{t}+R_{i}h_{t-1}+b_{i}\right)$$

(4) $$f_{t}=\sigma_{g}\left(W_{f}X_{t}+R_{f}h_{t-1}+b_{f}\right)$$

(5) $$g_{t}=\sigma_{c}\left(W_{g}X_{t}+R_{g}h_{t-1}+b_{g}\right)$$

(6) $$o_{t}=\sigma_{g}\left(W_{o}X_{t}+R_{o}h_{t-1}+b_{o}\right)$$

In the equations, *W_i_*, *W_f_*, *W_g_*, and *W_o_* are the input weights in the gates, $\sigma_{g}$ is the sigmoid equation, and $\sigma_{c}$ is the hyperbolic tangent function as the activation equation of state. *R_i_*, *R_f_*, *R_g_*, and *R_o_* are the recurrent weights in the gates, and *b_i_*, *b_f_*, *b_g_*, and *b_o_* are the biases. The input gate, encompassing the cell gate, serves to delineate which values from the input data are to be refreshed within the memory state. Concurrently, the forget gate is tasked with identifying which data should be omitted, whereas the output gate is responsible for determining the composition of the final hidden state. The cell and hidden states at subsequent steps over time can be represented by the following Equations (7) and (8), where ⊙ denotes the Hadamard product:

(7) $$c_{t}=f_{t}⊙c_{t-1}+i_{t}⊙g_{t}$$

(8) $$h_{t}=o_{t}⊙\sigma_{t}\left(c_{t}\right)$$

Modeling was conducted using code in MATLAB (R2023a), and for training options, optimization was achieved not by accumulating gradients at a constant rate but by updating gradients using an exponential moving average, employing Root Mean Square Propagation (RMS Prop). The LSTM layer was set to 64, with a maximum of 200 epochs. The learning rate was set to 0.001 for the first 0 to 100 epochs and adjusted to 0.0001 for the next 100 to 200 epochs.

### 2.3. Evaluation of the LSTM RNN Vibration Prediction Algorithm and AVC System

To evaluate the modeled algorithms and the AVC (active vibration canceling) system, vehicle vibration data from Seoul roads and the highway at 40 km/h, labeled as Seoul 40 and Highway 40, respectively, were replicated for 5 s due to the maximum data-saving time of the laser displacement sensor. The vibrations generated by the algorithm and those canceled by the AVC system, based on the inverse of the predicted vibration, were measured for displacement using a laser sensor.

To quantitatively assess each non-stationary vibration, the standard deviation (SD) of each dataset of results and the root mean squared error value at epoch 200 were calculated according to Equations (9) and (10). Accuracy and attenuation rates were determined based on the measured vibrations of Seoul 40 and Highway 40 as benchmarks before and after AVC implementation, respectively.

(9) $$SD=\sqrt{\frac{\sum_{i=1}^{n}{\left(x_{i}-\bar{x}\right)}^{2}}{n}}$$

(10) $$RMSE=\sqrt{\overset{n}{\underset{i=1}{\sum }}{\frac{\left({\hat{y}}_{i}-y_{i}\right)}{n}}^{2}}$$

## 3. Results

### 3.1. LSTM RNN Prediction Vibration Algorithm

**Figure 5:**
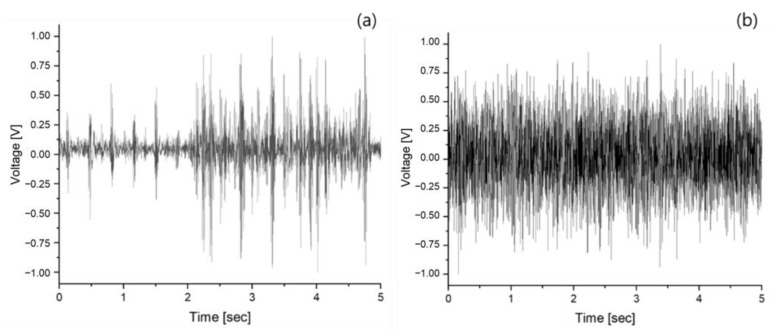
The normalized input voltage data of vibrations for (**a**) Seoul 40 and (**b**) Highway 40.

**Figure 6:**
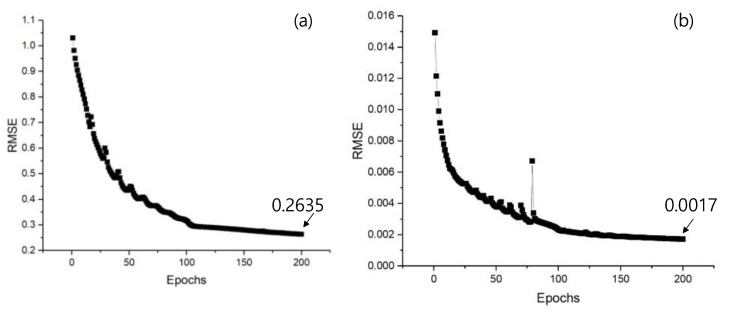
The RMSE results of LSTM RNN vibration prediction algorithms for (**a**) Seoul 40 and (**b**) Highway 40.

The normalized vibration datasets of Seoul 40 and Highway 40, created using actual measured acceleration data to develop the LSTM RNN vibration prediction algorithm and evaluate the AVC system, were represented as shown in Figure 5. The root mean squared error (RMSE) values at epoch 200 were 0.2635 for Seoul 40 and 0.0017 for Highway 40, as shown in Figure 6.

### 3.2. The Results of Vibrations and Evaluation of the AVC System 

**Figure 7:**
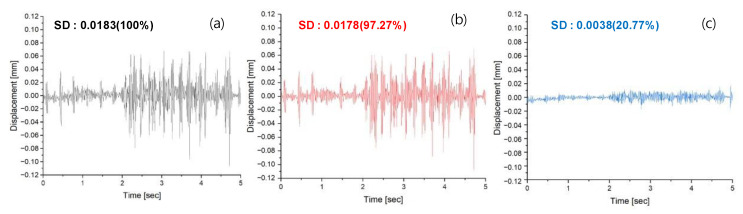
The displacement results of Seoul 40’s vibrations (**a**) as measured, (**b**) as predicted by the LSTM RNN algorithm, and (**c**) after the AVC system.

The measurement of actual vibrations of Seoul 40, the predicted vibrations using LSTM RNN, and the attenuated vibrations through the AVC system revealed in Figure 7 showed vibrations occurring between 2 and 4 s, which corresponded to the actual condition of manholes or road surfaces. The measured vibrations ranged from a maximum of 0.6774 mm to −1.0726 mm and after the implementation of the AVC system, the vibrations were measured to range from 0.1901 mm to −0.2029 mm. It was observed that the LSTM RNN predicted 97.27% of the vibrations measured by comparing SDs of Seoul 40 and predicted values accurately, while the vibrations when the AVC system was activated decreased significantly to 20.77%.

**Figure 8:**
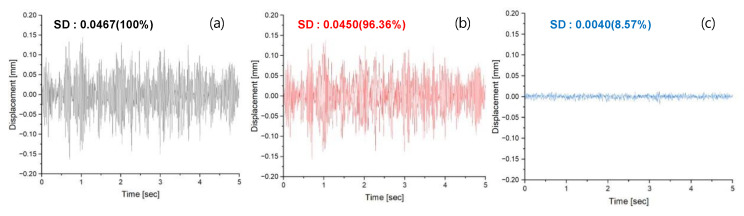
The displacement results of Highway 40’s vibrations (**a**) as measured, (**b**) as predicted by the LSTM RNN algorithm, and (**c**) after the AVC system.

Figure 8 depicts a graph of simulated vibration results for Highway 40. The measured size of actual vibrations ranged from 0.1450 mm to −0.1654 mm, and unlike Seoul 40, there were no significant differences in vibration occurrence. Analyzed through standard deviations and means, 96.36% of the measured vibrations were predicted. It was observed that the vibrations canceled by the AVC system amounted to 8.57% of the initial vibrations, indicating significant attenuation.

## 4. Discussion

Upon analyzing the results of the RMSE of the LSTM RNN vibration prediction algorithm modeled based on normalized vibration data, it was observed that until epoch 100 with a learning rate of 0.001, the RMSE values decreased significantly, indicating effective model training. Additionally, from epoch 101 to 200, it was noted that the RMSE values continued to decrease steadily with a learning rate of 0.0001, indicating more stable error reduction. Furthermore, despite both signals being normalized, Seoul 40 exhibited a higher RMSE value compared to Highway 40. This discrepancy was attributed to significant variations in vibration occurring during the process of storing and updating the LSTM RNN’s cell state, particularly in segments where vibrations differed markedly.

When comparing the graph of the reproduced vibration of Seoul 40 measured by a laser displacement sensor with the graph of the normalized input voltage, it was evident that there was a significant change in vibration approximately 2 s later. This observation indicated that the vibration reproduction using MFC had been successful. Furthermore, through a comparison of the waveform and SD values between the vibration of Seoul 40 and the predicted vibration graph generated by the LSTM RNN model, it was determined that the algorithm predicted accurately with a confidence level of 97.27%. Additionally, it was noted that the algorithm’s large RMSE value could be attributed to variations in vibration across different road conditions while driving.

The analysis of the SD values based on the confirmed vibration offsetting by the AVC system revealed that it was possible to offset 20.77% of Seoul 40’s vibrations. However, even after offsetting, it was observed that there were segments in the offset vibrations where the magnitude differed from the original vibrations. The results for Highway 40 showed an SD value of 0.0467, with the algorithm accurately predicting at 96.36%, while the AVC yielded 8.57%. However, when comparing the quantitative SD values of Seoul 40 and Highway 40 after offsetting by the AVC system, the difference was not significant, with an only 1.05 times difference compared to the initial vibration standard of 2.55 times. This is because, based on previous studies, it was revealed that the principle of the AVC system is to offset vibrations through counter-phase vibrations rather than damping and absorbing vibrations. Therefore, it can offset vibrations below a certain amplitude, regardless of the target vibration, as indicated in previous research.

This study confirms that the AVC system is capable of offsetting not only periodic sine wave vibrations, as in previous studies, but also actual non-periodic vibrations under various road conditions. Additionally, it achieved the development of an accurate LSTM RNN vibration prediction algorithm crucial for efficient vibration offsetting.

## 5. Conclusions

In this study, we have successfully developed and evaluated an active vibration canceling (AVC) system that utilizes macro-fiber composites (MFC) and an advanced AI algorithm based on long short-term memory (LSTM) to predict and cancel non-periodic vibrations in vehicle interiors during driving. The system demonstrates a novel integration of smart material technology and machine learning to address the challenge of improving ride comfort and reducing the adverse effects of road-induced vibrations.

The experimental results confirm the AVC system’s capability to significantly attenuate real-world, non-periodic vibrations canceled during vehicle operation on both urban (Seoul) and highway road conditions. Through the deployment of MFC sensors and actuators, the system could detect vibrations and generate counter-phase vibrations to effectively reduce the amplitude of the detected vibrations. The LSTM-based vibration prediction algorithm played a crucial role in this process, offering high accuracy in predicting the occurrence and characteristics of incoming vibrations, thus allowing for timely and effective vibration cancellation.

In conclusion, this research provides a promising approach to mitigating vehicle interior vibrations, leveraging the synergistic potential of smart materials and artificial intelligence. The findings of this study could pave the way for more sophisticated and integrated solutions in the field of vibration control, with broad implications for automotive engineering, smart material applications, and AI-driven control systems.

## Figures and Tables

**Table 1:** The mechanical and electronical properties of the MFC d31 type.

Properties	Symbol	MFC d31 Type [19]
Young’s modulus [GPa]	Y11 (fiber direction)	30.3
	Y33 (electrode direction)	15.9
Shear modulus [GPa]	G12	5.5
Piezoelectric constant [pC/N or pm/V]	d_31_	−0.017
Poisson’s ratio	$v_{12}$	0.31
	$v_{21}$	0.16
Density [g/${\mathrm{cm}}^{3}$]	ρ	0.544
Operation voltage range [V]	-	−60~360
Sensing frequency range [Hz]	-	0~1 M
Actuating frequency range [Hz]	-	0~10 k

## Data Availability

Data are contained within the article.
